# National Trends and Risk Factors for Dysphagia After Anterior Cervical Discectomy and Fusion

**DOI:** 10.1002/lary.70588

**Published:** 2026-04-27

**Authors:** Caryn J. Ha, Sraavya G. Anne, Sara Morgan, Fadar Oliver Otite, Kenneth Yan

**Affiliations:** ^1^ Department of Otolaryngology Head & Neck Surgery Rutgers New Jersey Medical School Newark New Jersey USA; ^2^ Department of Neurosurgery Rutgers New Jersey Medical School Newark New Jersey USA

**Keywords:** anterior cervical discectomy and fusion, dysphagia, national trends, risk factors, surgical indications

## Abstract

**Background:**

Dysphagia remains one of the most common complications following anterior cervical discectomy and fusion (ACDF), yet recent national trends and risk factors across surgical indications have not been fully characterized.

**Objective:**

To evaluate national trends in ACDF and dysphagia rates and to identify patient, surgical, and indication‐specific risk factors for postoperative dysphagia.

**Methods:**

A retrospective analysis of the Nationwide Inpatient Sample identified adult patients undergoing elective ACDF from 2016 to 2022. Multivariable logistic regression was used to determine independent risk factors for dysphagia and develop a predictive nomogram. Separate models were also constructed for single‐ and multilevel ACDF.

**Results:**

Among 496,425 hospitalizations, dysphagia occurred in 7.7% of patients. Despite a 57.3% decline in ACDF, the odds of dysphagia increased at an estimated 11.4% annually (OR: 1.11, 95% CI: 1.09–1.13; *p* < 0.001). Cervical diffuse idiopathic skeletal hyperostosis (OR: 5.41; 95% CI: 3.81–7.67), cervical spine fracture (OR: 1.73; 95% CI: 1.31–2.30), and pseudoarthrosis (OR: 1.36; 95% CI: 1.15–1.61) were strong independent predictors of dysphagia. Additional risk factors included racial minority status, higher comorbidity burden, and care at urban teaching hospitals. Risk factors differed between single‐ and multilevel ACDF. Dysphagia was associated with higher rates of complications, including aspiration pneumonia, percutaneous endoscopic gastrostomy placement, and tracheostomy (all *p* < 0.001).

**Conclusions:**

These findings represent the first national analysis to demonstrate a decline in inpatient ACDF volumes yet rising postoperative dysphagia rates. Selective surgical indications emerged as key predictors, informing the development of a nomogram for risk stratification and preoperative optimization in ACDF patients.

**Level of Evidence:**

3.

## Graphical abstract

From 2016 to 2023, inpatient anterior cervical discectomy and fusion (ACDF) volume declined 52.2% while postoperative dysphagia rates increased 11.4% annually. Analysis of 496,425 cases demonstrated that dysphagia risk varied markedly by surgical indication, with cervical diffuse idiopathic skeletal hyperostosis, cervical spine fracture, and pseudoarthrosis emerging as the strongest independent predictors, alongside multilevel surgery and greater comorbidity burden. These findings informed the development of a predictive nomogram and underscore the need for individualized perioperative risk stratification in ACDF candidates.

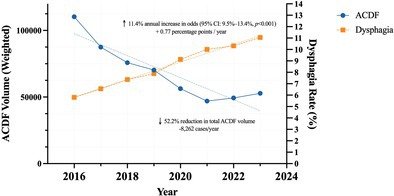

## 1 Background

Anterior cervical discectomy and fusion (ACDF) is among the most common surgical interventions for cervical spine disorders [1], with approximately 132,000 procedures performed annually in the United States [2]. Despite its overall favorable outcomes, postoperative dysphagia remains a frequent complication, affecting up to 60% of patients [3, 4, 5, 6, 7]. Oropharyngeal dysphagia may result from retropharyngeal and prevertebral soft tissue edema, direct pharyngeal irritation, or recurrent laryngeal nerve injury, leading to sequelae such as aspiration [8], increased mortality [9], and reduced quality of life [10, 11].

Reported risk factors for postoperative dysphagia in ACDF include revision surgery, multilevel approaches, prolonged operative time, and the use of recombinant human bone morphogenetic protein [9, 12, 13, 14, 15, 16, 17, 18, 19]. However, most prior studies are limited by small sample sizes or single‐institution cohorts [12, 14, 15]. While some studies leveraged the National Inpatient Sample (NIS), the largest publicly available all‐payer inpatient care database in the United States [20], they predominantly analyzed ACDF as a single, homogeneous cohort or examined only a narrow range of surgical indications [16, 18, 21, 22]. This represents a critical limitation, as indications such as cervical disc herniation or kyphotic deformity involve distinct pathologies with unique anatomical and technical considerations that may differentially impact dysphagia risk. Furthermore, although multilevel approaches have consistently been shown to increase dysphagia risk [12, 14, 15, 16, 17, 18, 23], it remains unclear whether specific risk factors differ between single‐ and multilevel procedures.

With evolving surgical techniques and expanding indications for ACDF [1, 3], along with the adoption of ICD‐10 coding enabling more precise classification of cervical spine pathologies, we conducted an updated, large‐scale analysis on recent national trends and risk factors for dysphagia following ACDF, stratified by primary surgical indication. We hypothesize that both the prevalence and predictors of postoperative dysphagia vary significantly by indication, and that single‐ and multilevel ACDF have distinct risk profiles.

## 2 Materials and Methods

### 2.1 Study Design and Setting

This retrospective study was conducted in accordance with the Strengthening the Reporting of Observational Studies in Epidemiology (STROBE) reporting guidelines and utilized data from the NIS, maintained by the Agency for Healthcare Research and Quality as part of the Healthcare Cost and Utilization Project (HCUP) [20]. The NIS employs a stratified sampling design that represents over 7 million hospitalizations annually across 49 participating states and jurisdictions [24]. All diagnoses and procedures were coded using the International Classification of Diseases, Clinical Modification (ICD‐10‐CM) and Procedure Coding System (ICD‐10‐PCS) to ensure consistency following its nationwide implementation in October 2015 [25]. The ICD‐10‐CM/PCS codes used in this study are provided in Supporting Table 1.

### 2.2 Eligibility Criteria

Adult (≥ 18 years) elective ACDF hospitalizations from 2016 to 2022 were identified in the NIS database. Primary analyses were restricted to 2016–2022, with 2023 data used only for select trend analyses due to differences in available covariates. Patients were excluded if they had nonelective ACDF to reduce confounding from traumatic or emergent indications, or if they had concurrent posterior cervical decompression and fusion.

Additionally, patients with dysphagia listed in the primary diagnosis field were excluded to omit those with preexisting or admitting diagnosis of dysphagia. Dysphagia across all subtypes (unspecified, oral, oropharyngeal, pharyngeal, pharyngoesophageal) occurring during the index hospitalization was identified using ICD‐10‐CM codes (Supporting Table 1). Subgroup analyses were performed based on primary surgical indication, including cervical spondylosis, cervical disc herniation, cervical disc degeneration, cervical stenosis, cervical ossification of the posterior longitudinal ligament (OPLL), cervical diffuse idiopathic skeletal hyperostosis (DISH), cervical kyphosis, cervical spine fracture, cervical dislocation, cervical spinal cord injury (SCI), pseudoarthrosis, rheumatoid arthritis (RA), and ankylosing spondylitis (AS). Spinal tumors were identified as a surgical indication but were excluded due to their low frequency and risk of model instability.

### 2.3 Outcomes

The primary outcome was postoperative dysphagia. Secondary outcomes included additional conditions associated with dysphagia, such as vocal cord paralysis, upper aerodigestive tract injuries, as well as complications from dysphagia, including aspiration pneumonia, esophageal perforation, and procedural interventions, including percutaneous endoscopic gastrostomy (PEG), tracheostomy, and mechanical ventilation.

### 2.4 Data Management and Missing Data

All statistical analyses were conducted using Stata/SE version 19.5 (StataCorp LLC, College Station, Texas, USA, 2025). Missing data across covariates was minimal (3.11%) and handled using complete‐case analysis without imputation.

### 2.5 Quantitative Variables and Statistical Methods

Demographic and clinical characteristics were summarized using nationally weighted estimates in accordance with the HCUP Data User Agreement. Annual US population estimates from the US Census Bureau were used to calculate procedure rates per 100,000 population. All monetary values were reported in US dollars (USD). Continuous variables were reported as means ± standard deviations, and categorical variables as frequencies (percentages). Group differences were assessed using Pearson's *χ* ^2^ test for categorical variables and the Student's *t*‐test for continuous variables. For univariate analyses, Bonferroni correction was applied to *p*‐values to account for multiple comparisons.

Two multivariable logistic regression models were constructed. Model 1 identified independent risk factors of dysphagia following ACDF, and Model 2 evaluated whether these risk factors differed between single‐level and multilevel ACDF. All models were adjusted for patient demographics (age, sex, race), insurance status, hospital characteristics (teaching status, location), year, and comorbidities using the Elixhauser Comorbidity Index (ECI) [26]. To ensure model stability, pairwise co‐occurrence between surgical indications was quantified using the Jaccard index (range: 0 = no co‐occurrence to 1 = perfect co‐occurrence) [27]. Based on observed statistical co‐occurrence, cervical spondylosis, disc herniation, disc degeneration, and stenosis were grouped together as “cervical degenerative disorders,” while all other indications were retained as separate variables. Multicollinearity was evaluated using variance inflation factors (VIFs). All covariates demonstrated VIFs < 2, except hospital teaching status (VIF = 5.96), which was retained for clinical relevance. For multivariable analyses, *p*‐values were adjusted using the Benjamini‐Hochberg procedure to control the false discovery rate. All tests were two‐sided, with *p*‐values < 0.05 considered statistically significant.

### 2.6 Sensitivity Analysis

To assess potential confounding by indication, patients with cervical DISH, a known cause of preoperative dysphagia, were excluded in a separate analysis. To account for potential preoperative dysphagia, patients with both dysphagia and malnutrition coded within the first 10 diagnosis fields were excluded, as the co‐occurrence of these diagnoses was thought to represent malnutrition secondary to chronic dysphagia rather than a new postoperative event. Multivariable regression models were re‐estimated to assess the robustness of effect estimates.

### 2.7 Nomogram

Select variables that were statistically significant in Model 1 were included in a predictive nomogram. Patients without cervical DISH, pseudoarthrosis, or cervical spine fracture were categorized as “0,” and overlapping indications (*n* = 19) were excluded for model stability. Calibration was assessed by the Hosmer–Lemeshow goodness‐of‐fit test. Discrimination was assessed using the concordance index (C‐index) and the area under the receiver operating characteristic curve (AUC) with 95% confidence intervals. Internal validation was performed using 200 bootstrap resamples. The nomogram model was made using R software with relevant packages (version 4.5.1, R Foundation for Statistical Computing).

### 2.8 Ethical Considerations

This study received an exemption from the Rutgers Institutional Review Board (IRB #: Pro2025002153) as a nonhuman subject study. All patient data were deidentified and publicly available; therefore, informed consent was waived under 45 CFR 46.104(d) (4).

## 3 Results

### 3.1 National Trends in ACDF and Dysphagia Rates

**FIGURE 1:**
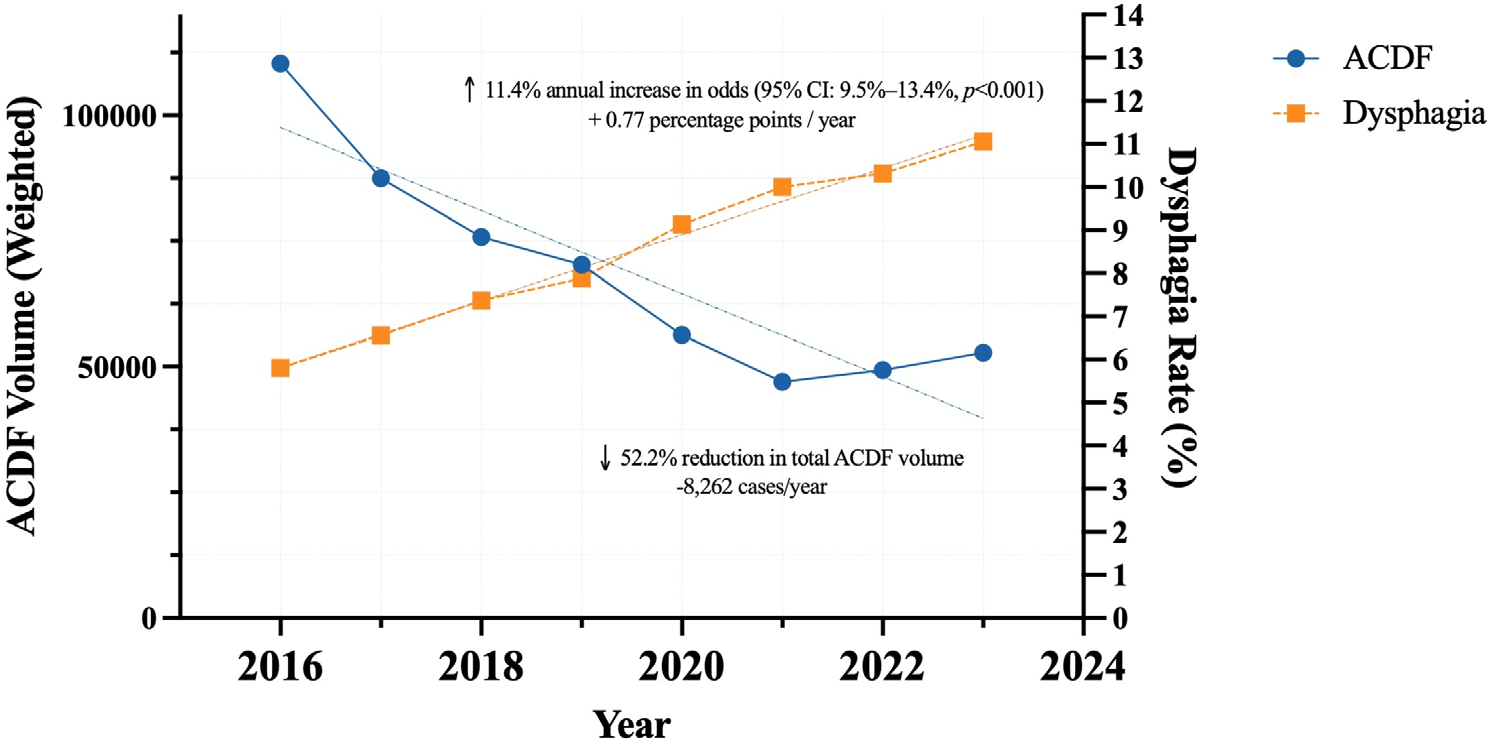
National Trends in Adult Elective Inpatient Anterior Cervical Discectomy and Fusion (ACDF) Procedures and Dysphagia Rates by Weighted Estimates in the United States, 2016–2023. Trends in weighted national estimates of annual elective ACDF volume (left *Y*‐axis, blue) and dysphagia rates (right *Y*‐axis, orange) from 2016 to 2023 are displayed using dual *Y*‐axes for visual comparison. Dashed lines represent simple linear regression fits. The odds of dysphagia increased annually by 11.4% (CI: 9.5%–13.4%; *p* < 0.001), corresponding to an absolute increase of 0.77 percentage points per year (CI: 0.69–0.85, *p* < 0.001). [Color figure can be viewed in the online issue, which is available at www.laryngoscope.com]

Between 2016 and 2023, there were 549,195 (weighted national estimates) adult elective inpatient ACDF cases, of which 44,010 (8.0%) had dysphagia. The rate of dysphagia rose from 5.8% in 2016 to 11.1% in 2023, corresponding to an estimated 11.4% annual increase in the odds of dysphagia (OR: 1.11, CI: 1.09–1.13, *p* < 0.001) or an absolute increase of 0.77 percentage points per year (CI 0.69–0.85, *p* < 0.001). During the same period, the national rate of elective inpatient ACDF declined from 44.2 to 18.9 procedures per 100,000 US population between 2016 and 2022, representing a 57.3% decline. This downward trend was followed by a modest increase to 20.0 procedures per 100,000 in 2023, up 6.0% from 2022. From 2016 to 2023, overall ACDF volume declined by an average of 8262 cases per year (CI: 4734‐11,790, *p* = 0.0012), representing a 52.2% reduction in total volume (Figure 1, Supporting Table 2).

To assess whether changes in patient selection contributed to the decline in ACDF volume, a subgroup analysis was performed on patients with the most common cervical spine conditions treated with ACDF. The prevalence estimates per 100,000 US population remained stable across these indications, and the proportion of patients undergoing surgery demonstrated minimal year‐to‐year variation, suggesting that the decline in ACDF was not primarily driven by a shift toward nonoperative management (Supporting Figure 1). Instead, the decline in ACDF volume from 2016 to 2023 closely paralleled a rise in cervical disc replacement (CDR) and posterior cervical foraminotomy (PCF) cases (Supporting Figure 2). Correlation analysis demonstrated a strong inverse relationship between ACDF volume and CDR/PCF utilization across indications, with Pearson coefficients ranging from −0.93 to −0.97 (*p* < 0.001) (Supporting Figure 1).

### 3.2 Patient Demographics and Characteristics

In the primary analytic cohort, 496,425 (weighted national estimates) adult elective inpatient ACDF cases from 2016 to 2022 were identified, of which 38,180 (7.7%) had dysphagia. (Supporting Figure 3). Demographic and clinical characteristics stratified by dysphagia status are presented in Table 1. Patients with dysphagia were older (61.2 ± 11.6 vs. 57.7 ± 11.6 years, *p* < 0.001), more often male (48.83% vs. 47.56%, *p* < 0.05), and had Medicare coverage (45.97% vs. 36.04%, *p* < 0.001) compared to those without dysphagia. Dysphagia was also associated with greater comorbidity burden (ECI Score: 2.29 ± 1.72 vs. 1.77 ± 1.52, *p* < 0.001) but lower smoking prevalence (13.17% vs. 16.20%, *p* < 0.001). Additional comorbidity comparisons are provided in Supporting Table 3. Patients with dysphagia were more often receiving care at urban teaching centers (78.73% vs. 73.01%, *p* < 0.001), had longer hospitalizations (3.95 ± 4.58 vs. 1.66 ± 2.12 days, *p* < 0.001), were less likely to be discharged home (68.62% vs. 86.16%, *p* < 0.001), and more likely to require home health care (17.56% vs. 9.51%, *p* < 0.001) or SNF placement (13.14% vs. 3.90%, *p* < 0.001). Although in‐hospital mortality was low overall, patients with dysphagia had a higher mortality rate (0.20% vs. 0.08%, *p* < 0.01). Dysphagia was also more frequent among patients undergoing multilevel compared to single‐level ACDF (76.39% vs. 69.07%, *p* < 0.001) (Table 1).

**TABLE 1:** Demographic characteristics among adults undergoing elective inpatient anterior cervical discectomy and fusion (ACDF) in the United States, 2016–2022.

Category	Total (ACDF)	Dysphagia	No dysphagia	*p* ^a^
Number of admissions (unweighted | weighted) (*N* [%])	99,285 | 496,425 (100%)	7636 | 38,180 (7.70%)	91,649 | 458,245 (92.31%)	—
Age (Mean ± SD)	58.01158 ± 11.59667	61.17522 ± 11.63687	57.74799 ± 11.55435	< 0.001
Gender				**< 0.05**
Female	52.35%	51.17%	52.44%	
Male	47.65%	48.83%	47.56%	
Race				**< 0.001**
White	78.00%	74.18%	78.32%	
Black	10.30%	13.47%	10.04%	
Hispanic	6.97%	7.81%	6.90%	
Asian or Pacific Islander	1.54%	1.62%	1.53%	
Native American	0.52%	0.66%	0.50%	
Other	2.67%	2.27%	2.70%	
Insurance type				**< 0.001**
Medicare	36.81%	45.97%	36.04%	
Medicaid	10.01%	9.89%	10.02%	
Private Insurance	43.14%	35.75%	43.76%	
Self‐pay	1.11%	0.91%	1.13%	
No Charge	0.07%	0.13%	0.07%	
Other	8.86%	7.36%	8.98%	
Hospital Region				**< 0.001**
Northeast	17.74%	15.10%	17.96%	
Midwest	19.64%	20.22%	19.60%	
South	42.30%	41.37%	42.38%	
West	20.32%	23.31%	20.07%	
Hospital teaching status				**< 0.001**
Rural	3.63%	2.79%	3.70%	
Urban nonteaching	22.92%	18.48%	23.29%	
Urban teaching	73.45%	78.73%	73.01%	
Length of stay (Mean ± SD)	1.840736 ± 2.473953	3.9522 ± 4.575003	1.664808 ± 2.117603	**< 0.001**
Total charge in USD (Mean ± SD)	$85,247.92 ± $67,799.62	$112,706.8 ± $90,215.62	$82,968.01 ± $65,076.67	**< 0.001**
Smoking				**< 0.001**
Yes	15.97%	13.17%	16.20%	
No	84.03%	86.83%	83.80%	
Number of operative levels				**< 0.001**
1	30.37%	23.61%	30.93%	
> 1	69.63%	76.39%	69.07%	
Elixhauser Comorbidity Index (ECI) score (Mean ± SD)	1.809286 ± 1.543795	2.291645 ± 1.716117	1.769097 ± 1.521686	**< 0.001**
0	22.26%	14.69%	22.89%	**< 0.001**
1–5	75.45%	80.72%	75.01%	
6+	2.29%	4.58%	2.10%	

*Note:* Bolded values indicate statistical significance.

Abbreviations: ACDF: Anterior Cervical Discectomy and Fusion, ECI: Elixhauser Comorbidity Index, N/A: missing data, SD: Standard deviation, SNF: Skilled Nursing Facility, USD: United States Dollar.

^a^ Bonferroni‐adjusted *p*‐values from *χ* ^2^ tests comparing patients with and without dysphagia.

### 3.3 Complications of ACDF and Post‐Operative Dysphagia

There were several known complications of ACDF associated with higher dysphagia rates (Table 2). Patients with dysphagia had higher rates of vocal cord paralysis (0.81% vs. 0.10%, *p* < 0.001), including both unilateral (0.54% vs. 0.06%, *p* < 0.001) and bilateral paralysis (0.09% vs. 0.00%, *p* < 0.001). Upper aerodigestive tract injuries involving the pharynx, larynx, cervical esophagus, or trachea were also more common in the dysphagia group (0.03% vs. 0.00%, *p* < 0.05), as well as esophageal perforation (0.10% vs. 0.01%, *p* < 0.001). Additionally, patients with dysphagia experienced higher rates of aspiration pneumonia (2.88% vs. 0.24%, *p* < 0.001), and increased need for interventions such as PEG placement (3.42% vs. 0.07%, *p* < 0.001), tracheostomy (0.56% vs. 0.07%, *p* < 0.001), and mechanical ventilation (3.84% vs. 1.04%, *p* < 0.001).

**TABLE 2:** Complications among adults undergoing elective inpatient ACDF in the United States, 2016–2022.

Category	Total ACDF (%)	Dysphagia (% of column)	No dysphagia (% of column)	*p* ^a^
Paralysis (any type) of VC and Larynx	0.15%	0.81%	0.10%	**< 0.001**
Unilateral paralysis of VC and Larynx	0.10%	0.54%	0.06%	**< 0.001**
Bilateral Paralysis of VC and Larynx	0.01%	0.09%	0.00%	**< 0.001**
Upper Aerodigestive Tract (Pharynx, Cervical Esophagus, Trachea, or Larynx) Injuries	0.01%	0.04%	0.00%	**< 0.05**
Esophageal Perforation	0.02%	0.10%	0.01%	**< 0.001**
Aspiration Pneumonia	0.44%	2.88%	0.24%	**< 0.001**
PEG	0.32%	3.42%	0.07%	**< 0.001**
Tracheostomy	0.11%	0.56%	0.07%	**< 0.001**
Mechanical Ventilation	1.25%	3.84%	1.04%	**< 0.001**

*Note:* Percentages represent the distribution of specified complications within each column (Total ACDF, dysphagia, no dysphagia). Bolded values indicate statistical significance.

Abbreviations: ACDF: anterior cervical discectomy and fusion, PEG: percutaneous endoscopic gastrostomy, VC: vocal cords.

^a^ Bonferroni‐adjusted *p*‐values from *χ* ^2^ tests comparing patients with and without dysphagia.

### 3.4 Primary Surgical Indications

Thirteen primary surgical indications were identified to categorize the underlying pathology for patients undergoing ACDF, capturing 97.9% of elective cases. These included cervical spondylosis, cervical disc herniation, cervical disc degeneration, cervical stenosis, cervical OPLL, cervical DISH, cervical kyphosis, cervical spine fracture, cervical dislocation, cervical SCI, pseudoarthrosis, RA, and AS.

The most common indications were cervical stenosis (64.93%), cervical disc herniation (53.48%), and cervical spondylosis (44.40%), collectively representing the majority of procedures. Less frequent indications included cervical disc degeneration (5.23%), rheumatoid arthritis (2.77%), pseudoarthrosis (1.96%), cervical dislocation (0.62%), cervical OPLL (0.57%), cervical spine fracture (0.57%), cervical kyphosis (0.26%), cervical SCI (0.26%), cervical DISH (0.16%), and ankylosing spondylitis (0.15%) (Supporting Table 4).

**FIGURE 2:**
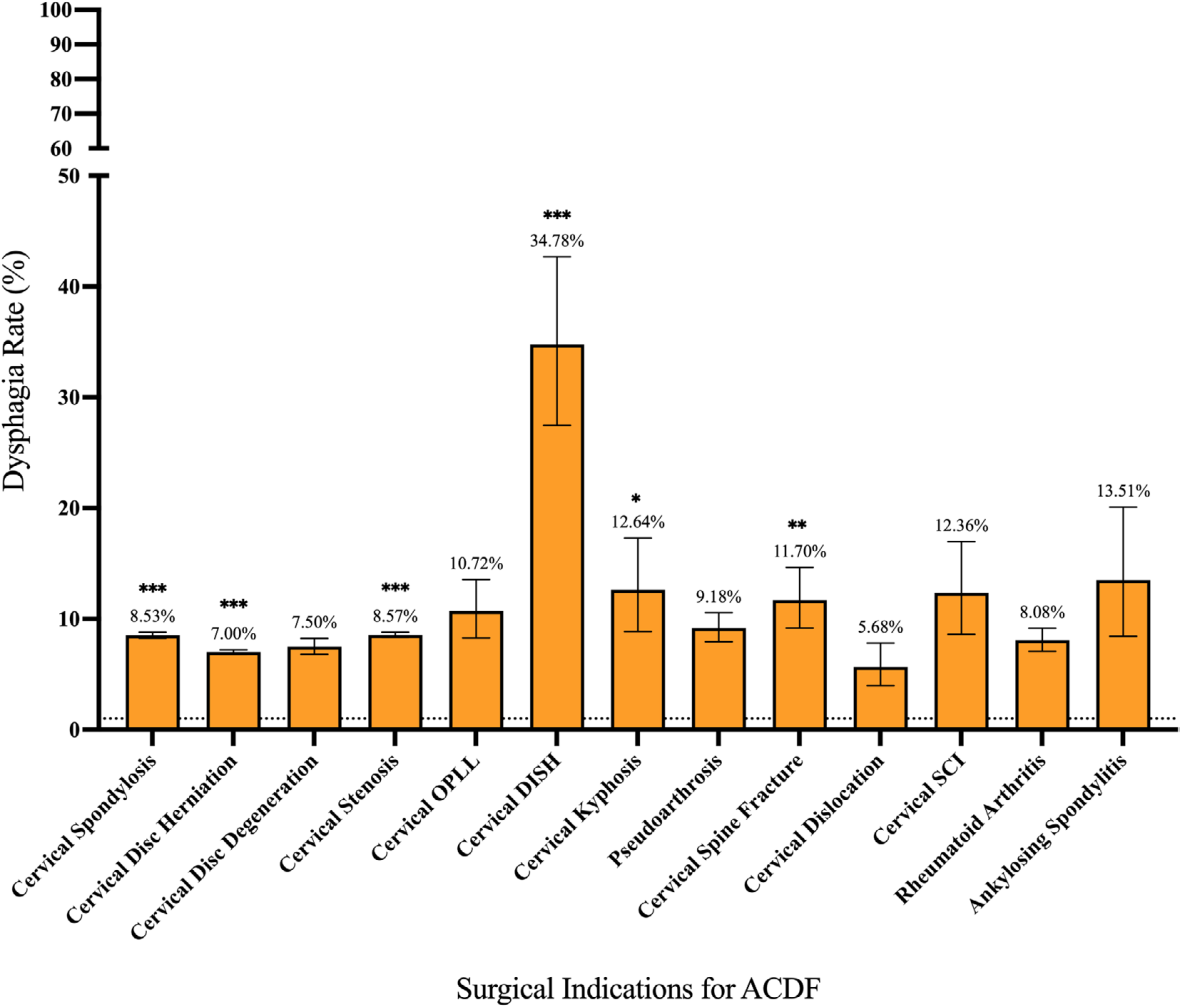
Dysphagia rates among adult patients undergoing elective inpatient anterior cervical discectomy and fusion (ACDF) by cervical spine condition in the United States, 2016–2022. Bar graph of dysphagia rates (%) among ACDF patients stratified by surgical indication. Bars represent point estimates of dysphagia rates, and error bars represent 95% confidence intervals calculated for proportions. Significance markers reflect Bonferroni‐adjusted *p*‐values from comparisons between patients with and without dysphagia for each surgical indication (*p < 0.05, **p < 0.01, ***p < 0.001). ACDF: anterior cervical discectomy and fusion. DISH: diffuse idiopathic skeletal hyperostosis. SCI: spinal cord injury. [Color figure can be viewed in the online issue, which is available at www.laryngoscope.com]

Dysphagia rates varied substantially by surgical indication (Figure 2, Supporting Table 4). The highest dysphagia rate was observed among patients with cervical DISH (34.78%, *p* < 0.001), followed by cervical kyphosis (12.64%, *p* < 0.05), cervical SCI (12.36%, *p* > 0.05), and cervical spine fracture (11.70%, *p* < 0.01). Patients undergoing ACDF for more common degenerative conditions such as cervical stenosis (8.57%), cervical spondylosis (8.53%), and cervical disc herniation (7.00%) also demonstrated associations with dysphagia (all *p* < 0.001).

### 3.5 Co‐Occurrence Patterns of Surgical Indications

**FIGURE 3:**
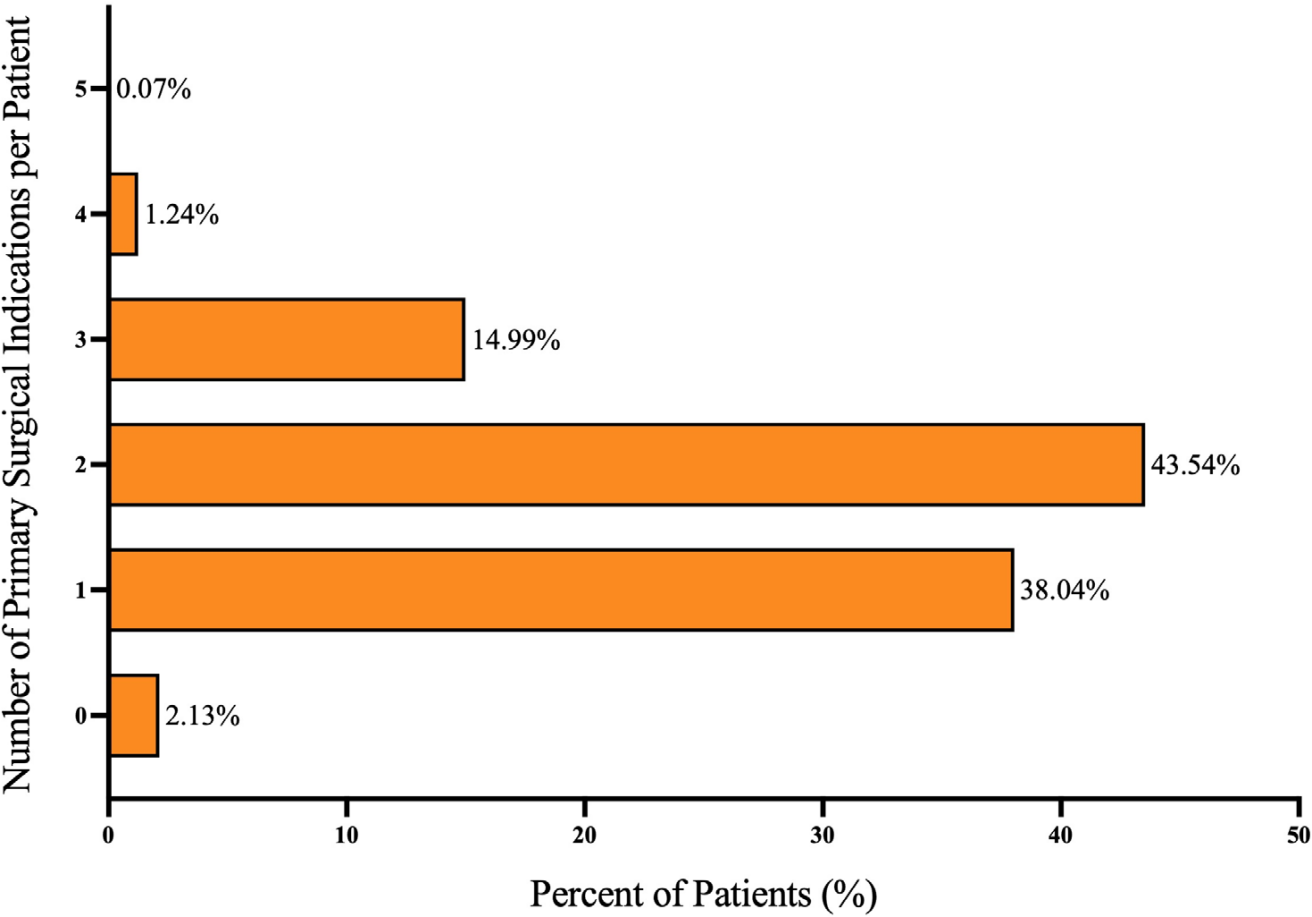
Distribution of primary surgical indication counts among anterior cervical discectomy and fusion (ACDF) patients. *Y*‐axis represents the total number of primary surgical indications identified per patient, ranging from 0 to a maximum of 5 co‐occurring indications. The *x*‐axis represents the proportion of patients within each category relative to the total study population. [Color figure can be viewed in the online issue, which is available at www.laryngoscope.com]

The majority of patients presented with multiple concurrent surgical indications for ACDF. Approximately 43.54% of patients had two indications, 14.99% had three, 1.24% had four, and 0.07% had five indications. Only 38.04% of patients had a single surgical indication (Figure 3).

Among patients with exclusively two indications, the most frequent diagnostic combinations were cervical disc herniation with cervical stenosis (42.92%), cervical spondylosis with cervical stenosis (32.85%), cervical spondylosis with cervical disc herniation (13.54%), and cervical stenosis with cervical disc degeneration (3.05%). For patients with three indications, the most frequent diagnostic combinations were cervical spondylosis with cervical disc herniation and cervical stenosis (71.15%), cervical spondylosis with cervical disc degeneration and cervical stenosis (5.62%), and cervical disc herniation with cervical disc degeneration and cervical stenosis (4.61%).

Pairwise co‐occurrence analysis across all patients demonstrated distinct phenotypic patterns among patients undergoing ACDF. Degenerative pathologies, including cervical spondylosis, disc herniation, disc degeneration, and stenosis, had the strongest co‐occurrence patterns, with high Jaccard similarity indices ranging from 0.319 to 0.550. In contrast, cervical kyphosis (Jaccard index < 0.021) and pseudoarthrosis (Jaccard index < 0.024) most often occurred in isolation. Similarly, inflammatory conditions such as AS and RA (Jaccard index < 0.038), traumatic indications including cervical spine fracture, dislocation, and SCI (Jaccard index < 0.041), and ossification disorders such as cervical DISH and OPLL (Jaccard index < 0.017) demonstrated largely distinct, nonoverlapping patterns (Supporting Figure 4).

### 3.6 Multivariable Risk Factors for Dysphagia

The first multivariable regression model (Model 1) assessed for independent risk factors for dysphagia in patients who underwent ACDF (Table 3). Independent predictors included cervical DISH (OR: 5.41, CI: 3.81–7.67), cervical spine fracture (OR: 1.73, CI: 1.31–2.30), and pseudoarthrosis (OR: 1.36, CI: 1.15–1.61). Given the known association between cervical DISH and preoperative dysphagia, a sensitivity analysis was performed, excluding patients with cervical DISH to investigate confounding by indication. The effect estimates remained consistent with Model 1 (Supporting Table 5), demonstrating robustness of the observed associations.

**TABLE 3:** Model 1: multivariable logistic regression analysis of independent risk factors for dysphagia following ACDF in the United States, 2016–2022.

Variable	OR (95% CI)	*p* ^a^
Age	1.02 (1.01–1.02)	**< 0.001**
Female sex	0.96 (0.91–1.01)	> 0.05
Year	1.08 (1.06–1.10)	**< 0.001**
Race		
Black	1.43 (1.32–1.55)	**< 0.001**
Hispanic	1.20 (1.09–1.33)	**< 0.01**
Asian or Pacific Islander	1.03 (0.85–1.26)	> 0.05
Native American	1.38 (1.02–1.87)	> 0.05
Other	0.98 (0.83–1.15)	> 0.05
Elixhauser Comorbidity Index		
1–5 comorbidities	1.35 (1.24–1.47)	**< 0.001**
6+ comorbidities	2.20 (1.87–2.60)	**< 0.001**
Other comorbidities		
Chronic Pulmonary Disease	1.05 (0.98–1.12)	> 0.05
Hypertension	1.02 (0.96–1.08)	> 0.05
Obesity	1.03 (0.96–1.10)	> 0.05
Diabetes	1.10 (1.03–1.16)	> 0.05
Smoking	0.84 (0.78–0.91)	**< 0.001**
Surgery level		
Multilevel ACDF	1.34 (1.26–1.42)	**< 0.001**
Primary surgical indication		
Cervical Degenerative Disorders	1.01 (0.87–1.18)	> 0.05
Cervical OPLL	1.23 (0.91–1.67)	> 0.05
Cervical DISH	5.41 (3.81–7.67)	**< 0.001**
Cervical Kyphosis	1.57 (1.08–2.29)	> 0.05
Pseudoarthrosis	1.36 (1.15–1.61)	**< 0.01**
Cervical Spine Fracture	1.73 (1.31–2.30)	**< 0.001**
Cervical Dislocation	0.88 (0.60–1.29)	> 0.05
Cervical SCI	1.52 (1.04–2.23)	> 0.05
Rheumatoid Arthritis	0.87 (0.75–1.01)	> 0.05
Ankylosing Spondylitis	1.15 (0.67–1.97)	> 0.05
Insurance		
Medicaid	1.08 (0.97–1.19)	> 0.05
Private insurance	0.88 (0.83–0.95)	**< 0.01**
Self‐pay	0.82 (0.62–1.08)	> 0.05
No charge	1.93 (0.71–5.26)	> 0.05
Other	0.87 (0.78–0.97)	**< 0.05**
Hospital Teaching Status		
Urban nonteaching	1.18 (0.98–1.41)	> 0.05
Urban teaching	1.54 (1.30–1.82)	**< 0.001**
Hospital Location		
Midwest	1.18 (1.04–1.35)	**< 0.05**
South	1.09 (0.96–1.24)	> 0.05
West	1.32 (1.17–1.50)	**< 0.001**

*Note:* Bolded values indicate statistical significance.

Abbreviations: ACDF: anterior cervical discectomy and fusion, CI: confidence interval, DISH: cervical diffuse idiopathic skeletal hyperostosis, OPLL: cervical ossification of the posterior longitudinal ligament, OR: odds ratio, SCI: cervical spinal cord injury.

^a^ Comparisons between patients with and without dysphagia were conducted using multivariable logistic regression with *p*‐values adjusted for multiple testing using Benjamini‐Hochberg false discovery rate (FDR) method.

Additional risk factors for dysphagia included Black (OR: 1.43, CI: 1.32–1.55) and Hispanic (OR: 1.20, CI: 1.09–1.33) race. Increasing comorbidity burden was also associated with higher risk (1–5 comorbidities; OR: 1.35, CI: 1.24–1.47; 6+ comorbidities; OR: 2.20, CI: 1.87–2.60), as well as treatment at urban teaching hospitals (OR: 1.54, CI: 1.30–1.82). Undergoing multilevel ACDF was also associated with dysphagia (OR: 1.34, CI: 1.26–1.42). Smoking was associated with a lower risk of dysphagia (OR: 0.84, CI: 0.78–0.91). After identifying multilevel ACDF and comorbidities as independent predictors of dysphagia in multivariable models, we examined their annual trends from 2016 to 2022. The national rise in dysphagia closely paralleled increases in the proportion of multilevel procedures and patients with multiple comorbidities among ACDF cases (Supporting Figure 5). To account for potential preoperative dysphagia, a sensitivity analysis excluding patients with dysphagia coded among the first diagnostic code entries was conducted, and the analysis was repeated excluding concomitant malnutrition, assuming it likely represented a sequela of preexisting dysphagia. All independent predictors in Model 1 retained significance in both analyses (Supporting Tables 6 and 7).

The second multivariable regression model (Model 2) evaluated whether risk factors for dysphagia differed between single‐level and multilevel ACDF procedures (Table 4). Cervical DISH emerged as the strongest predictor of dysphagia regardless of surgical extent, with a stronger association observed in multilevel ACDF (OR: 6.05, CI: 4.08–8.98) compared to single‐level ACDF (OR: 3.80, CI: 1.77–8.15). Pseudoarthrosis (OR: 1.54, CI: 1.17–2.02) was a risk factor in the single‐level ACDF cohort only, whereas cervical spine fracture (OR: 1.82, CI: 1.22–2.73) was a risk factor in the multilevel cohort only. Additionally, private insurance was associated with lower odds of dysphagia across both single‐level and multilevel ACDF procedures.

**TABLE 4:** Model 2: multivariable logistic regression analysis of independent risk factors for dysphagia by ACDF surgical complexity in the United States, 2016–2022.

Variable	Single‐level ACDF		Multilevel ACDF	
	OR (95% CI)	*p* ^a^	OR (95% CI)	*p* ^a^
Age	1.02 (1.01–1.03)	**< 0.001**	1.02 (1.01–1.02)	**< 0.001**
Female sex	1.00 (0.90–1.11)	> 0.05	0.95 (0.90–1.01)	> 0.05
Year	1.13 (1.10–1.16)	**< 0.001**	1.07 (1.05–1.09)	**< 0.001**
Race				
Black	1.45 (1.25–1.68)	**< 0.001**	1.43 (1.30–1.56)	**< 0.001**
Hispanic	1.30 (1.08–1.57)	**< 0.05**	1.17 (1.04–1.31)	**< 0.05**
Asian or Pacific Islander	1.31 (0.92–1.85)	> 0.05	0.93 (0.74–1.18)	> 0.05
Native American	0.55 (0.22–1.35)	> 0.05	1.69 (1.22–2.33)	**< 0.01**
Other	0.91 (0.65–1.30)	> 0.05	1.00 (0.83–1.20)	> 0.05
Elixhauser Comorbidity Index				
1–5 comorbidities	1.36 (1.14–1.61)	**< 0.01**	1.34 (1.21–1.48)	**< 0.001**
6+ comorbidities	2.42 (1.75–3.35)	**< 0.001**	2.12 (1.76–2.57)	**< 0.001**
Other comorbidities				
Chronic Pulmonary Disease	1.07 (0.94–1.21)	> 0.05	1.04 (0.97–1.11)	> 0.05
Hypertension	1.02 (0.90–1.16)	> 0.05	1.02 (0.95–1.10)	> 0.05
Obesity	0.98 (0.86–1.11)	> 0.05	1.04 (0.96–1.13)	> 0.05
Diabetes	1.13 (1.00–1.28)	> 0.05	1.08 (1.01–1.16)	> 0.05
Smoking	0.83 (0.71–0.96)	> 0.05	0.85 (0.78–0.92)	**< 0.001**
Primary surgical indication				
Cervical degenerative disorders	0.86 (0.67–1.09)	> 0.05	1.13 (0.93–1.37)	> 0.05
Cervical OPLL	1.86 (0.98–3.54)	> 0.05	1.13 (0.81–1.58)	> 0.05
Cervical DISH	3.80 (1.77–8.15)	**< 0.01**	6.05 (4.08–8.98)	**< 0.001**
Cervical Kyphosis	1.55 (0.35–6.95)	> 0.05	1.59 (1.08–2.34)	> 0.05
Pseudoarthrosis	1.54 (1.17–2.02)	**< 0.05**	1.24 (1.00–1.52)	> 0.05
Cervical Spine Fracture	1.55 (1.04–2.32)	> 0.05	1.82 (1.22–2.73)	**< 0.01**
Cervical Dislocation	0.65 (0.32–1.34)	> 0.05	1.01 (0.66–1.53)	> 0.05
Cervical SCI	2.16 (1.06–4.43)	> 0.05	1.39 (0.89–2.16)	> 0.05
Rheumatoid Arthritis	0.69 (0.50–0.95)	> 0.05	0.93 (0.79–1.09)	> 0.05
Ankylosing Spondylitis	0.93 (0.31–2.76)	> 0.05	1.22 (0.66–2.23)	> 0.05
Insurance				
Medicaid	1.19 (0.99–1.42)	> 0.05	1.03 (0.92–1.16)	> 0.05
Private insurance	0.83 (0.72–0.94)	**< 0.05**	0.90 (0.83–0.97)	**< 0.05**
Self‐pay	0.49 (0.26–0.91)	> 0.05	0.95 (0.71–1.27)	> 0.05
No charge	0.89 (0.10–7.89)	> 0.05	2.20 (0.75–6.49)	> 0.05
Other	0.89 (0.72–1.10)	> 0.05	0.85 (0.75–0.97)	> 0.05
Hospital teaching status				
Urban nonteaching	1.03 (0.73–1.46)	> 0.05	1.23 (1.00–1.51)	> 0.05
Urban teaching	1.36 (0.98–1.88)	> 0.05	1.61 (1.33–1.94)	**< 0.001**
Hospital location				
Midwest	1.21 (1.01–1.46)	> 0.05	1.18 (1.02–1.36)	> 0.05
South	1.23 (1.03–1.46)	> 0.05	1.05 (0.92–1.20)	> 0.05
West	1.42 (1.18–1.71)	**< 0.01**	1.29 (1.12–1.49)	**< 0.01**

*Note:* Bolded values indicate statistical significance.

Abbreviations: ACDF: anterior cervical discectomy and fusion, CI: confidence interval, DISH: cervical diffuse idiopathic skeletal hyperostosis, OPLL: cervical ossification of the posterior longitudinal ligament, OR: odds ratio, SCI: cervical spinal cord injury.

^a^ Comparisons between patients with and without dysphagia were conducted using multivariable logistic regression with *p*‐values adjusted for multiple testing using Benjamini‐Hochberg false discovery rate (FDR) method.

**FIGURE 4:**
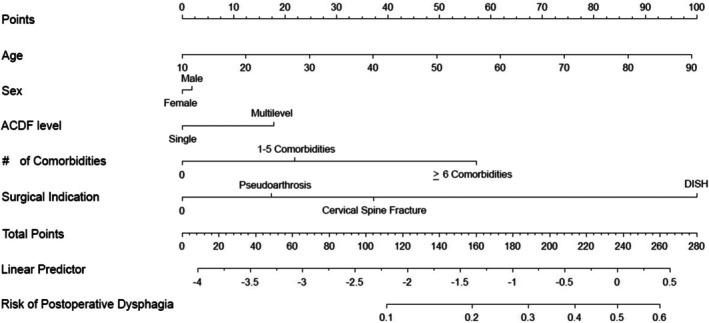
Clinical nomogram for predicting postoperative dysphagia risk in anterior cervical discectomy and fusion (ACDF). Each predictor variable (age, sex, ACDF level, number of comorbidities, and surgical indication) is assigned a point value by drawing a vertical line upward to the “Points” scale. The sum of these points is located on the “Total Points” axis, which corresponds to the predicted probability of postoperative dysphagia shown on the “Risk of Postoperative Dysphagia” scale. ACDF: anterior cervical discectomy and fusion. DISH: diffuse idiopathic skeletal hyperostosis.

A predictive nomogram was developed based on variables that remained significant in the multivariable logistic regression Model 1 (Figure 4). Predictors included age, sex, ACDF level (single vs. multilevel), number of Elixhauser comorbidities (1–5 vs. ≥ 6), and surgical indication (pseudoarthrosis, cervical spine fracture, DISH, or none). Each factor was assigned a weighted point value according to its relative contribution to postoperative dysphagia risk.

The nomogram demonstrated modest discriminatory ability between patients with and without postoperative dysphagia (AUC = 0.61, 95% CI: 0.60–0.62, SE = 0.0033) and good overall accuracy (Brier score = 0.070, 95% CI: 0.069–0.071). The Hosmer–Lemeshow goodness‐of‐fit test showed good calibration and agreement between the predicted and observed probabilities (*χ* ^2^ = 12.12, df = 8, *p* = 0.15). Internal bootstrap validation with 200 resamples revealed minimal optimism (bias = 0.00055) (Supporting Figure 6).

## 4 Discussion

In this first nationally representative analysis to stratify postoperative dysphagia risk after ACDF by both primary surgical indication and operative extent, we identified evolving trends in practice and outcomes. Despite a 57.3% decline in inpatient ACDF volume between 2016 and 2022, the rate of postoperative dysphagia increased 11.4% annually, reaching an overall rate of 7.7%. Cervical DISH, cervical spine fracture, and pseudoarthrosis were found to be independently associated with dysphagia risk, while smoking was associated with lower odds. Finally, our analysis revealed that risk factor profiles differ between single‐ and multilevel ACDF procedures, indicating that surgical complexity modifies the influence of individual risk factors. Using these factors, we developed a nomogram to estimate patient‐specific risk for postoperative dysphagia following ACDF.

### 4.1 National Trends in ACDF and Dysphagia Rates

The observed decline in total inpatient ACDF procedures likely reflects a broader shift toward outpatient surgery [28, 29, 30, 31, 32, 33], supported by growing evidence of comparable or improved outcomes and lower revision rates [34, 35, 36, 37, 38]. Further analysis of our cohort revealed that disease prevalence and the proportion of patients undergoing surgery remained stable over the study period; however, alternative procedures, such as CDR and PCF, increased, consistent with the findings by Ibrahim et al. [1]. Collectively, these trends suggest that the observed decline in ACDF procedures most likely reflects evolving surgical preferences rather than a true decrease in surgical intervention rates.

Our study found the overall dysphagia rate to be 7.7%, which is comparable to the 8.5% rate reported in a prior systematic review by Shriver et al. [39]. We also observed a paradoxical increase in dysphagia rates among patients who received ACDF. This may be due to both improved recognition of complications and changes in patient selection for inpatient procedures, as dysphagia following ACDF has been historically underdiagnosed [10, 40, 41, 42, 43, 44]. In addition, as lower‐risk patients are increasingly managed through outpatient ACDF, the remaining inpatient cohort may represent older patients with greater comorbidity and surgical complexity, all risk factors known to elevate postoperative complications [45, 46, 47, 48]. Consistent with this, our analysis found that from 2016 to 2022, the proportion of multilevel ACDF and comorbidity burden among inpatient cases increased nationwide. Together, these factors likely contribute to the paradoxical observed rise in postoperative dysphagia rates despite a decline in inpatient ACDF volume.

### 4.2 Risk Factors for Dysphagia: Patient Demographics and Characteristics

In multivariable regression analyses adjusting for demographic and clinical covariates, we found older age, race, higher comorbidity burden, and treatment at urban teaching hospitals were risk factors for postoperative dysphagia. Our findings on the association between age [16, 45, 46], race [18], and comorbidity burden [18, 49, 50] with dysphagia are consistent with prior studies. The higher risk at urban teaching hospitals may be capturing referral patterns for more complex, medically vulnerable patient populations, though further study is needed to confirm this.

Interestingly, our analysis found that active smoking was associated with lower odds of dysphagia. However, this is unlikely to represent a true protective effect. Potential explanations include underreporting of smoking status, altered pharyngeal sensory thresholds leading to an underrecognition of dysphagia [51], or selection bias in elective surgical populations, where patients may be advised to stop smoking preoperatively.

### 4.3 Risk Factors for Dysphagia: Primary Surgical Indications

Although ACDF is performed for a diverse range of pathologies, stratification by primary surgical indication showed that most patients presented with only one or two indications. This suggests that postoperative dysphagia is largely driven by a small set of dominant, high‐risk indications rather than being evenly distributed across all ACDF indications. Specifically, cervical DISH was among the rarest indications (0.16%) but had the highest rate of postoperative dysphagia (34.78%) and odds across all regression models, aligning with prior literature identifying it as a high‐risk pathology [52, 53, 54, 55]. Although preoperative dysphagia may present in DISH, our sensitivity analyses yielded nearly identical effect estimates to the main analysis, suggesting that our findings reflect true postoperative risk. Even without symptomatic dysphagia, bulky anterior cervical hyperosteophytosis can disrupt normal tissue planes and reduce the compliance of adjacent structures [54, 56]. Chung et al. demonstrated that narrowing of the prevertebral soft tissue correlates with both pre‐ and postoperative dysphagia severity and may predict dysphagia relapse [57]. Furthermore, ACDF for DISH includes concomitant osteophytectomy for decompression and intraoperative exposure, introducing additional local tissue manipulation. These factors may compound preexisting soft‐tissue vulnerability, predisposing to postoperative edema and retropharyngeal swelling, which are thought to be associated with dysphagia after ACDF [39, 40, 58]. Consistent with this, a recent systematic review by Malhotra et al. demonstrated that postoperative dysphagia rates following osteophytectomy for DISH were comparable to ACDF [59]. This underscores how, regardless of surgical intervention or hardware placement, the intrinsic pathology of DISH may predispose patients to postoperative dysphagia.

We observed a larger effect size for cervical DISH as a risk factor in the multilevel ACDF group compared to single‐level. Because DISH is defined as ossification spanning ≥ 4 contiguous vertebrae (or ≥ 3 consecutive levels), it inherently requires multilevel surgery and longer operative times, both established risk factors for postoperative dysphagia [8, 18, 60]. Similarly, cervical spine fracture was associated with postoperative dysphagia in the unstratified regression model but retained significance only in the multilevel ACDF subgroup likely due to fracture stabilization requiring greater soft tissue exposure and longer operative times as well. Additional risk in cervical spine fractures may also come from local injury to nearby structures or edema related to the initial trauma. In contrast, single‐level procedures are less common and generally require less aggressive exposure and retraction, which may explain the absence of significance in the single‐level cohort.

Pseudoarthrosis remained a significant risk factor across the main regression model and for single‐level ACDF. This underscores its role as a marker for revision ACDF surgery, which often entails extensive scar tissue dissection and prior hardware removal. These technical demands may increase the risk of esophageal and recurrent laryngeal nerve injury. However, prior studies have been inconsistent regarding revision status as a dysphagia risk factor, with some reporting a significant association [52] and others finding no effect [15, 61, 62]. A recent study by Cochran et al. also found pseudoarthrosis associated with higher odds of dysphagia (OR 1.73 [95% CI: 1.70–1.81]) [63], suggesting that revision status may only be clinically relevant to postoperative dysphagia risk when performed for nonunion rather than other causes.

### 4.4 Nomogram

We developed a nomogram based on the independent predictors identified in our multivariable models, including age, comorbidity burden, ACDF complexity, and relevant primary surgical indications. While the nomogram demonstrated good calibration, its discriminative ability was modest, likely reflecting the multifactorial nature of dysphagia risk that includes intraoperative factors and surgical techniques beyond patient characteristics alone. Nonetheless, the model offers a practical and clinically accessible tool for identifying high‐risk patients who may benefit from enhanced perioperative counseling and postoperative management.

### 4.5 Limitations

Several limitations should be acknowledged. As a retrospective analysis, this study is subject to biases, including underreporting, misclassification, and missing data. Additionally, the NIS database has inherent limitations, such as the absence of clinical details, including physical examination findings, operative time, intraoperative complications, and specifics on surgical technique (e.g., retractor use or anterior plating), all of which may influence dysphagia risk. Temporal information on diagnoses, chronic conditions, and comorbidities is also unavailable, limiting the ability to establish clear causal relationships. Although patients with a primary diagnosis of dysphagia were excluded and sensitivity analyses were performed, ICD coding does not allow differentiation between preoperative, transient, or postoperative dysphagia and may lead to misclassification. Moreover, NIS captures admissions rather than individual patients, precluding longitudinal follow‐up, which may underestimate dysphagia developing beyond discharge or overestimate readmission‐related complications. Finally, this study is limited to inpatient ACDF procedures and may not be generalizable to outpatient settings. Future studies incorporating both inpatient and outpatient data are warranted. Despite these limitations, multivariable analyses were performed to adjust for confounding factors, and this study represents the largest nationwide analysis examining contemporary trends and granular dysphagia risk factors following ACDF.

### 4.6 Conclusion

In this large‐scale, nationally representative analysis, we stratified postoperative dysphagia risk by both primary surgical indication and operative complexity. We found that specific pathologies carry disproportionately higher dysphagia risk, and the magnitude of this risk varies with the number of levels fused. These findings indicate that postoperative dysphagia is shaped not only by surgical extent but also by the underlying indication. By integrating patient‐level and procedure‐specific risk factors, our work provides a framework for more precise risk prediction, patient selection, and perioperative optimization to reduce postoperative morbidity.

## Funding

The authors have nothing to report.

## Ethics Statement

The NIS is a de‐identified administrative database exempt from the Institutional Review Board informed consent requirements.

## Consent

The authors have nothing to report.

## Conflicts of Interest

The authors declare no conflicts of interest.

## Supporting information


**Supporting Figure 1.** Pearson Correlation Coefficient on Top Cervical Surgical Indications (2016–2023).
**Supporting Figure 2**. National Trends of Inpatient Cervical Surgeries in the United States, 2016–2023.
**Supporting Figure 3**. Primary analytic cohort selection for elective anterior cervical discectomy and fusion (ACDF) using the National Inpatient Sample, 2016–2022.
**Supporting Figure 4**. Jaccard similarity heatmap of surgical indication co‐occurrence in patients undergoing anterior cervical discectomy and fusion (ACDF).
**Supporting Figure 5**. Dysphagia trends and associated risk factors in anterior cervical discectomy and fusion (ACDF) patients.
**Supporting Figure 6**. Nomogram model performance for predicting postoperative dysphagia.
**Supporting Table 1**. Coding definitions for variables.
**Supporting Table 2**. National trends in elective ACDF and dysphagia rates in the United States, 2016–2023.
**Supporting Table 3**. Comorbidities among adults undergoing elective inpatient anterior cervical discectomy and fusion (ACDF) in the United States, 2016–2022.
**Supporting Table 4**. Primary surgical indications and dysphagia rates for elective ACDF in the United States, 2016–2022.
**Supporting Table 5**. Sensitivity analysis: exclusion of patients with cervical diffuse idiopathic skeletal hyperostosis (DISH).
**Supporting Table 6**. Sensitivity analysis: exclusion of patients with concurrent dysphagia and malnutrition in early diagnosis fields.
**Supporting Table 7**. Sensitivity analysis: exclusion of patients with dysphagia only in early diagnosis fields.

## Data Availability

The data that support the findings of this study are available on request from the corresponding author. The data are not publicly available due to privacy or ethical restrictions.
