# Supplementary material for: National Trends and Risk Factors for Dysphagia After Anterior Cervical Discectomy and Fusion
Source: Laryngoscope. 2026 Apr 27;136(9):3918–31. doi: 10.1002/lary.70588 (PMC13460480; doi:10.1002/lary.70588)
Supplement: Supplementary file 1 [file LARY-136-3918-s001.docx]

|  |  |
| --- | --- |
|  |  |
|  |  |

**Supporting Figure 1. Pearson Correlation Coefficient on Top Cervical Surgical Indications (2016-2023)**

Stacked bar graphs (left panel) demonstrate the annual proportion of patients undergoing anterior cervical discectomy and fusion (ACDF), posterior cervical foraminotomy (PCF) / cervical disc replacement (CDR), or no surgery for cervical spondylosis (top) and cervical disc herniation (bottom). Scatter plots (right panel) illustrate the inverse correlation between annual ACDF and PCF/CDR utilization.

**Supporting Figure 2. National Trends of Inpatient Cervical Surgeries in the United States, 2016-2023**

Trends in weighted national estimates of annual elective anterior cervical discectomy and fusion (ACDF) volume (blue) compared with combined estimates of cervical disc replacement (CDR) and posterior cervical foraminotomy (PCF) (purple) from 2016 to 2023.

**Supporting Figure 3. Primary Analytic Cohort Selection for Elective Anterior Cervical Discectomy and Fusion (ACDF) using the National Inpatient Sample, 2016-2022**

Unweighted data from the National Inpatient Sample (NIS), 2016–2022, were used to identify adult patients (≥18 years old) who underwent elective anterior cervical decompression and fusion (ACDF). Patients were excluded if they underwent non-elective ACDF, concurrent posterior cervical decompression and fusion (PCDF), were under 18 years old, or were admitted with a primary diagnosis of dysphagia. The final cohort was stratified by dysphagia status.

**Supporting Figure 4. Jaccard Similarity Heatmap of Surgical Indication Co-Occurrence in Patients Undergoing Anterior Cervical Discectomy and Fusion (ACDF)**

DISH: Diffuse Idiopathic Skeletal Hyperostosis

OPLL: Ossification of Posterior Longitudinal Ligament

SCI: Spinal Cord Injury

RA: Rheumatoid Arthritis

AS: Ankylosing Spondylitis

Heatmap demonstrating pairwise co-occurrence patterns between primary surgical indications for ACDF, calculated using the Jaccard similarity index. Each cell reflects the proportion of patients with both indications relative to those with either indication. Red indicates stronger co-occurrence. Cells with perfect co-occurrence of 1 (diagonal) are indicated by white color.

**Supporting Figure 5. Dysphagia Trends and Associated Risk Factors in Anterior Cervical Discectomy and Fusion (ACDF) Patients**

Annual dysphagia rates and rates of associated risk factors among patients undergoing ACDF from 2016–2022. The proportion of multi-level ACDF procedures and patients receiving ACDF with 1-5 comorbidities are shown on the left Y-axis. Dysphagia rates and ≥ 6 comorbidities are plotted on the right Y-axis due to lower rates. All values reflect the proportion of total ACDF patients for each respective year.

**Supporting Figure 6. Nomogram model performance for predicting postoperative dysphagia.**

(A) Receiver Operating Curve (ROC) demonstrating the discrimination ability of the nomogram model for predicting postoperative dysphagia. (area under curve [AUC] 0.61, 95% CI 0.60-0.62)

(B) Calibration plot comparing predicted (x-axis) and observed (y-axis) probabilities of postoperative dysphagia across deciles of risk.

**Supporting Table 1: Coding Definitions for Variables**

| **Variable** | **ICD-10-CM / ICD-10-PCS** |
| --- | --- |
| Dysphagia | R13.1, R13.10, R13.11, R13.12, R13.13, R13.14, R13.19 |
| ACDF | 0RG10A0, 0RG1070, 0RG10J0, 0RG10K0, 0RG1370, 0RG13A0, 0RG13J0, 0RG13K0, 0RG1470, 0RG14A0, 0RG14J0, 0RG14K0, 0RG2070, 0RG20A0, 0RG20J0, 0RG20K0, 0RG2370, 0RG23A0, 0RG23J0, 0RG23K0, 0RG2470, 0RG24A0, 0RG24J0, 0RG24K0 |
| ACDF one-level | 0RG10A0, 0RG1070, 0RG10J0, 0RG10K0, 0RG1370, 0RG13A0, 0RG13J0, 0RG13K0, 0RG1470, 0RG14A0, 0RG14J0, 0RG14K0 |
| ACDF multilevel | 0RG2070, 0RG20A0, 0RG20J0, 0RG20K0, 0RG2370, 0RG23A0, 0RG23J0, 0RG23K0, 0RG2470, 0RG24A0, 0RG24J0, 0RG24K0 |
| PCDF | 0RG1071, 0RG107J, 0RG10A1, 0RG10AJ, 0RG10J1, 0RG10JJ, 0RG10K1, 0RG10KJ, 0RG1371, 0RG137J, 0RG13Z1, 0RG13ZJ, 0RG13A1, 0RG13AJ, 0RG13J1, 0RG13JJ, 0RG13K1, 0RG13KJ, 0RG1471, 0RG147J, 0RG14A1, 0RG14AJ, 0RG14J1, 0RG14JJ, 0RG14K1, 0RG14KJ, 0RG14Z1, 0RG14ZJ, 0RG2071, 0RG207J, 0RG20A1, 0RG20AJ, 0RG20J1, 0RG20JJ, 0RG20K1, 0RG20KJ, 0RG20Z1, 0RG20ZJ, 0RG2371, 0RG237J, 0RG23A1, 0RG23AJ, 0RG23J1, 0RG23JJ, 0RG23Z1, 0RG23ZJ, 0RG23K1, 0RG23KJ, 0RG2471, 0RG247J, 0RG24A1, 0RG24AJ, 0RG24J1, 0RG24JJ, 0RG24K1, 0RG24KJ, 0RG24Z1 |
| Cervical Spondylosis | M4721, M4722, M4723, M47811, M47812, M47813, M47891, M47892, M47893, M4711, M4712, M4713 |
| Cervical Disc Herniation | M5020, M5021, M5022, M5023 |
| Cervical Disc Disorder with Myelopathy or Radiculopathy | M5000, M5001, M5002, M5003, M5010, M5011, M5012, M5013 |
| Cervical Disc Disorder without Myelopathy or Radiculopathy | M5080, M5081, M5082, M5083 |
| Cervical Disc Degeneration | M5030, M5031, M5032, M5033 |
| Cervical Stenosis | M4801, M4802, M4803 |
| OPLL (cervical) | M488X1, M488X2, M488X3 |
| DISH (cervical) | M4811, M4812, M4813 |
| Cervical Kyphosis | M4003, M4012, M40202, M40292 |
| Pseudoarthrosis | M960 |
| Cervical Spine Fractures | S120, S121, S122, S123, S124, S125, S126, S128, S129 |
| Cervical Spine Dislocations | S131 |
| Cervical SCI | S140, S141, S1411, S1412, S1413, S1414 |
| Rheumatoid Arthritis | M05, M06 |
| Ankylosing Spondylitis | M45, M450, M451, M452, M453, M454, M455, M456, M457, M458, M459, M45A, M45A0, M45A1, M45A2, M45A3, M45A4, M45A5, M45A6, M45A7, M45A8, M45AB |
| Malnutrition | E40, E41, E42, E43, E44, E45, E46 |
| Aspiration pneumonia | J690 |
| Paralysis of vocal cords and larynx | J380 |
| Paralysis of vocal cords and larynx, unspecified | J3800 |
| Paralysis of vocal cords and larynx, unilateral | J3801 |
| Paralysis of vocal cords and larynx, bilateral | J3802 |
| Other specified injuries of pharynx and cervical esophagus | S1985 |
| Other specified injuries of cervical trachea | S1982 |
| Other specified injuries of larynx | S1981 |
| Esophageal Perforation | K223 |
| Percutaneous Endoscopic Gastrostomy | 0DH63UZ, 0DH64UZ, 0D164Z4 |
| Tracheostomy | 0B110F4, 0B110Z4, 0B114F4, 0B114Z4, 0B113F4, 0B113Z4, 0BH13EZ |
| Mechanical Ventilation | 5A09357, 5A09457, 5A09557, 5A1935Z, 5A1945Z, 5A1955Z |

**Supporting Table 2: National Trends in Elective ACDF and Dysphagia Rates in the United States, 2016-2023**

| **Year** | **Total (ACDF)*** | **Prevalence ⤈**  **(per 100,000)** | **With Dysphagia** | **Rate of Dysphagia** |
| --- | --- | --- | --- | --- |
| **2016** | 110,285 | 44.24 | 6,400 | 5.80% |
| **2017** | 87,470 | 34.79 | 5,740 | 6.56% |
| **2018** | 75,810 | 29.92 | 5,585 | 7.37% |
| **2019** | 70,285 | 27.54 | 5,535 | 7.88% |
| **2020** | 56,320 | 21.82 | 5,140 | 9.13% |
| **2021** | 46,965 | 18.12 | 4,695 | 10.00% |
| **2022** | 49,290 | 18.90 | 5,085 | 10.32% |
| **2023** | 52,770 | 20.02 | 5,830 | 11.05% |
| **All Years**  **(Total)** | 549,195 | 26.78 | 44,010 | 8.01% |
| *Weighted National Estimates  **^⤈^**Prevalence per 100,000 calculated using U.S. Census population data | | | | |

**Supporting Table 3: Comorbidities Among Adults Undergoing Elective Inpatient Anterior Cervical Discectomy and Fusion (ACDF) in the United States, 2016-2022**

| **Comorbidity** | **Total (ACDF)** | **Dysphagia** | **No Dysphagia** | ***p*-value^1^** |
| --- | --- | --- | --- | --- |
| **Number of admissions (**Unweighted \| Weighted) (**N[%])** | 99,285 \| 496,425 (100%) | 7,636 \| 38,180 (7.7%) | 91,649 \| 458,245 (92.3%) | -- |
| **Congestive Heart Failure** | 2.65% | 4.44% | 2.50% | **< 0.001** |
| **Cardiac Arrhythmias** | 6.25% | 11.01% | 5.85% | **< 0.001** |
| **Valvular Disease** | 1.94% | 3.01% | 1.85% | **< 0.001** |
| **Pulmonary Circulation Disorders** | 0.50% | 1.07% | 0.45% | **< 0.001** |
| **Peripheral Vascular Disorders** | 2.06% | 3.04% | 1.98% | **< 0.001** |
| **Hypertension, Uncomplicated** | 47.46% | 52.92% | 47.00% | **< 0.001** |
| **Hypertension, Complicated** | 5.45% | 8.94% | 5.15% | **< 0.001** |
| **Paralysis** | 1.03% | 2.44% | 0.91% | **< 0.001** |
| **Other Neurological Disorders** | 3.38% | 6.74% | 3.10% | **< 0.001** |
| **Chronic Pulmonary Disease** | 18.98% | 21.73% | 18.75% | **< 0.001** |
| **Diabetes, Uncomplicated** | 14.28% | 15.05% | 14.21% | >0.05 |
| **Diabetes, Complicated** | 6.99% | 12.24% | 6.55% | **< 0.001** |
| **Hypothyroidism** | 12.36% | 14.16% | 12.21% | **< 0.001** |
| **Renal Failure** | 4.46% | 6.91% | 4.26% | **< 0.001** |
| **Liver Disease** | 1.59% | 2.16% | 1.54% | **< 0.01** |
| **Peptic Ulcer Disease** | 0.25% | 0.46% | 0.23% | **< 0.01** |
| **AIDS/HIV** | 0.11% | 0.18% | 0.10% | >0.05 |
| **Lymphoma** | 0.17% | 0.21% | 0.17% | >0.05 |
| **Metastatic Cancer** | 0.19% | 0.46% | 0.16% | **< 0.001** |
| **Solid Tumor** | 0.44% | 0.71% | 0.42% | **< 0.05** |
| **Rheumatoid Arthritis/Collagen** | 4.14% | 4.64% | 4.10% | >0.05 |
| **Coagulopathy** | 1.35% | 2.48% | 1.26% | **< 0.001** |
| **Obesity** | 20.15% | 22.72% | 19.94% | **< 0.001** |
| **Weight Loss** | 0.41% | 1.96% | 0.28% | **< 0.001** |
| **Fluid/Electrolyte Disorders** | 3.70% | 9.94% | 3.18% | **< 0.001** |
| **Blood Loss Anemia** | 0.10% | 0.26% | 0.09% | **< 0.001** |
| **Deficiency Anemia** | 0.82% | 1.23% | 0.78% | **< 0.01** |
| **Alcohol Abuse** | 1.37% | 1.85% | 1.33% | **< 0.01** |
| **Drug Abuse** | 2.16% | 2.92% | 2.10% | **< 0.001** |
| **Psychoses** | 0.35% | 0.55% | 0.34% | >0.05 |
| **Depression** | 17.36% | 19.67% | 17.17% | **< 0.001** |
| **Malnutrition** | 0.19% | 1.09% | 0.12% | **< 0.001** |
| **Osteoporosis** | 0.00% | 0.00% | 0.00% | >0.05 |
| **Long-term Steroid Use** | 0.00% | 0.00% | 0.00% | >0.05 |
| **Opioid-related Disorder** | 0.00% | 0.00% | 0.00% | >0.05 |
| **Obstructive Sleep Apnea** | 0.00% | 0.00% | 0.00% | >0.05 |
| **GERD** | 0.00% | 0.00% | 0.00% | >0.05 |
| **Metabolic Syndrome** | 0.00% | 0.00% | 0.00% | >0.05 |
| GERD: Gastroesophageal Reflux Disease  Bolded values indicate statistical significance.  ^1^Bonferroni-adjusted *p*-values from χ² tests comparing patients with and without dysphagia | | | | |

**Supporting Table 4: Primary Surgical Indications and Dysphagia Rates for Elective ACDF in the United States, 2016-2022**

| **Condition** | **Number of Patients*** | **% of Total (ACDF*)** | **Dysphagia*** | **No Dysphagia*** | **Dysphagia Rates** | ***p*-value^1^** |
| --- | --- | --- | --- | --- | --- | --- |
| **Cervical Stenosis** | 322,340 | 64.93% | 27,620 | 58,944 | 8.57% | **<0.001** |
| **Cervical Disc Herniation** | 265,475 | 53.48% | 18,585 | 49,378 | 7.00% | **<0.001** |
| **Cervical Spondylosis** | 220,425 | 44.40% | 18,795 | 40,326 | 8.53% | **<0.001** |
| **Cervical Disc Degeneration** | 25,950 | 5.23% | 1,945 | 4,801 | 7.50% | >0.05 |
| **Rheumatoid Arthritis** | 13,745 | 2.77% | 1,109 | 2,527 | 8.08% | >0.05 |
| **Pseudoarthrosis** | 9,750 | 1.96% | 895 | 1,771 | 9.18% | >0.05 |
| **Cervical Dislocation** | 3,080 | 0.62% | 175 | 581 | 5.68% | >0.05 |
| **Cervical OPLL** | 2,845 | 0.57% | 305 | 508 | 10.72% | >0.05 |
| **Cervical Spine Fracture** | 2,820 | 0.57% | 330 | 498 | 11.70% | **<0.01** |
| **Cervical Kyphosis** | 1,305 | 0.26% | 165 | 228 | 12.64% | **<0.05** |
| **Cervical SCI** | 1,295 | 0.26% | 160 | 227 | 12.36% | >0.05 |
| **Cervical DISH** | 805 | 0.16% | 280 | 105 | 34.78% | **<0.001** |
| **Ankylosing Spondylitis** | 740 | 0.15% | 100 | 128 | 13.51% | >0.05 |
| *Weighted National Estimates  OPLL: Ossification of Posterior Longitudinal Ligament, SCI: Spinal Cord Injury, DISH: Diffuse Idiopathic Skeletal Hyperostosis  Bolded values indicate statistical significance.  ^1^Bonferroni-adjusted *p*-values from χ² tests comparing patients with and without dysphagia | | | | | | |

**Supporting Table 5:** Sensitivity Analysis: Exclusion of Patients with Cervical Diffuse Idiopathic Skeletal Hyperostosis (DISH)

| **Variable** | **OR (95% Confidence Interval)** | ***p*-value^1^** |
| --- | --- | --- |
| **Age** | 1.02 (1.01-1.02) | **<0.001** |
| **Female Sex** | 0.96 (0.92-1.01) | >0.05 |
| **Year** | 1.08 (1.07-1.10) | **<0.001** |
| **Race** | | |
| **Black** | 1.44 (1.33-1.56) | **<0.001** |
| **Hispanic** | 1.20 (1.09-1.33) | **<0.01** |
| **Asian or Pacific Islander** | 1.04 (0.85-1.27) | >0.05 |
| **Native American** | 1.38 (1.02-1.87) | >0.05 |
| **Other** | 0.98 (0.83-1.16) | >0.05 |
| **Elixhauser Comorbidity Index** | | |
| **1–5 comorbidities** | 1.32 (1.21-1.44) | **<0.001** |
| **6+ comorbidities** | 2.02 (1.71-2.39) | **<0.001** |
| **Other Comorbidities** | | |
| **Chronic Pulmonary Disease** | 1.06 (0.99-1.12) | >0.05 |
| **Hypertension** | 1.03 (0.96-1.09) | >0.05 |
| **Obesity** | 1.04 (0.97-1.11) | >0.05 |
| **Diabetes** | 1.10 (1.04-1.17) | **<0.01** |
| **Smoking** | 0.84 (0.78-0.90) | **<0.001** |
| **Surgery Level** | | |
| **Multilevel ACDF** | 1.33 (1.25-1.41) | **<0.001** |
| **Primary Surgical Indication** | | |
| **Cervical Degenerative Disorders** | 1.03 (0.88-1.20) | >0.05 |
| **Cervical OPLL** | 1.27 (0.93-1.72) | >0.05 |
| **Cervical Kyphosis** | 1.60 (1.10-2.32) | **<0.05** |
| **Pseudoarthrosis** | 1.37 (1.16-1.62) | **<0.01** |
| **Cervical Spine Fracture** | 1.74 (1.31-2.32) | **<0.001** |
| **Cervical Dislocation** | 0.87 (0.59-1.28) | >0.05 |
| **Cervical SCI** | 1.48 (1.00-2.19) | >0.05 |
| **Rheumatoid Arthritis** | 0.88 (0.76-1.02) | >0.05 |
| **Ankylosing Spondylitis** | 1.22 (0.72-2.09) | >0.05 |
| **Insurance** | | |
| **Medicaid** | 1.08 (0.98-1.19) | >0.05 |
| **Private insurance** | 0.88 (0.83-0.95) | **<0.01** |
| **Self-pay** | 0.82 (0.63-1.08) | >0.05 |
| **No charge** | 1.93 (0.71-5.26) | >0.05 |
| **Other** | 0.87 (0.78-0.97) | **<0.05** |
| **Hospital Teaching Status** | | |
| **Urban nonteaching** | 1.18 (0.98-1.42) | >0.05 |
| **Urban teaching** | 1.54 (1.30-1.83) | **<0.001** |
| **Hospital Location** | | |
| **Midwest** | 1.19 (1.05-1.36) | **<0.05** |
| **South** | 1.10 (0.97-1.25) | >0.05 |
| **West** | 1.34 (1.18-1.52) | **<0.001** |
| Footnote: This analysis excluded patients who had cervical diffuse idiopathic skeletal hyperostosis. Model covariates are identical to Model 1.  OR: Odds Ratio, CI: Confidence Interval, ACDF: Anterior Cervical Discectomy and Fusion, OPLL: Cervical Ossification of the Posterior Longitudinal Ligament, DISH: Cervical Diffuse Idiopathic Skeletal Hyperostosis, SCI: Cervical Spinal Cord Injury  ^1^Comparisons between patients with and without dysphagia were conducted using multivariable logistic regression with p-values adjusted for multiple testing using Benjamini-Hochberg false discovery rate (FDR) method | | |

**Supporting Table 6: Sensitivity Analysis: Exclusion of Patients with Concurrent Dysphagia and Malnutrition in Early Diagnosis Fields**

| **Variable** | **OR (95% Confidence Interval)** | ***p*-value^1^** |
| --- | --- | --- |
| **Age** | 1.02 (1.01-1.02) | **<0.001** |
| **Female Sex** | 0.96 (0.92-1.01) | >0.05 |
| **Year** | 1.08 (1.06-1.10) | **<0.001** |
| **Race** | | |
| **Black** | 1.43 (1.32-1.55) | **<0.001** |
| **Hispanic** | 1.19 (1.08-1.32) | **<0.01** |
| **Asian or Pacific Islander** | 1.04 (0.85-1.26) | >0.05 |
| **Native American** | 1.39 (1.03-1.88) | >0.05 |
| **Other** | 0.97 (0.82-1.15) | >0.05 |
| **Elixhauser Comorbidity Index** | | |
| **1–5 comorbidities** | 1.34 (1.23-1.46) | **<0.001** |
| **6+ comorbidities** | 2.17 (1.84-2.56) | **<0.001** |
| **Other Comorbidities** | | |
| **Chronic Pulmonary Disease** | 1.05 (0.98-1.12) | >0.05 |
| **Hypertension** | 1.02 (0.96-1.09) | >0.05 |
| **Obesity** | 1.03 (0.96-1.09) | >0.05 |
| **Diabetes** | 1.10 (1.03-1.17) | **<0.01** |
| **Smoking** | 0.84 (0.78-0.91) | **<0.001** |
| **Surgery Level** | | |
| **Multilevel ACDF** | 1.34 (1.26-1.42) | **<0.001** |
| **Primary Surgical Indication** | | |
| **Cervical Degenerative Disorders** | 1.02 (0.87-1.19) | >0.05 |
| **Cervical OPLL** | 1.24 (0.91-1.68) | >0.05 |
| **Cervical DISH** | 5.43 (3.83-7.71) | **<0.001** |
| **Cervical Kyphosis** | 1.58 (1.09-2.30) | **<0.05** |
| **Pseudoarthrosis** | 1.37 (1.15-1.62) | **<0.01** |
| **Cervical Spine Fracture** | 1.74 (1.31-2.31) | **<0.001** |
| **Cervical Dislocation** | 0.86 (0.58-1.26) | >0.05 |
| **Cervical SCI** | 1.53 (1.05-2.25) | >0.05 |
| **Rheumatoid Arthritis** | 0.87 (0.75-1.01) | >0.05 |
| **Ankylosing Spondylitis** | 1.16 (0.68-1.98) | >0.05 |
| **Insurance** | | |
| **Medicaid** | 1.08 (0.98-1.19) | >0.05 |
| **Private insurance** | 0.88 (0.83-0.95) | **<0.01** |
| **Self-pay** | 0.82 (0.63-1.08) | >0.05 |
| **No charge** | 1.94 (0.71-5.26) | >0.05 |
| **Other** | 0.86 (0.77-0.96) | **<0.05** |
| **Hospital Teaching Status** | | |
| **Urban nonteaching** | 1.17 (0.98-1.41) | >0.05 |
| **Urban teaching** | 1.53 (1.29-1.81) | **<0.001** |
| **Hospital Location** | | |
| **Midwest** | 1.18 (1.03-1.34) | **<0.05** |
| **South** | 1.09 (0.97-1.24) | >0.05 |
| **West** | 1.32 (1.16-1.50) | **<0.001** |
| Footnote: This analysis excluded patients who had both dysphagia and malnutrition codes documented within the first ten diagnosis fields at index admission. Model covariates are identical to Model 1.  OR: Odds Ratio, CI: Confidence Interval, ACDF: Anterior Cervical Discectomy and Fusion, OPLL: Cervical Ossification of the Posterior Longitudinal Ligament, DISH: Cervical Diffuse Idiopathic Skeletal Hyperostosis, SCI: Cervical Spinal Cord Injury  ^1^Comparisons between patients with and without dysphagia were conducted using multivariable logistic regression with p-values adjusted for multiple testing using Benjamini-Hochberg false discovery rate (FDR) method | | |

**Supporting Table 7: Sensitivity Analysis: Exclusion of Patients with Dysphagia Only in Early Diagnosis Fields**

| **Variable** | **OR (95% Confidence Interval)** | ***p*-value^1^** |
| --- | --- | --- |
| **Age** | 1.03 (1.03-1.04) | **<0.001** |
| **Female Sex** | 0.94 (0.85-1.05) | >0.05 |
| **Year** | 1.25 (1.22-1.29) | **<0.001** |
| **Race** | | |
| **Black** | 1.32 (1.13-1.54) | **<0.01** |
| **Hispanic** | 1.22 (1.00-1.48) | >0.05 |
| **Asian or Pacific Islander** | 0.91 (0.59-1.40) | >0.05 |
| **Native American** | 1.17 (0.58-2.33) | >0.05 |
| **Other** | 0.93 (0.65-1.34) | >0.05 |
| **Elixhauser Comorbidity Index** | | |
| **1–5 comorbidities** | 6.64 (4.58-9.61) | **<0.001** |
| **6+ comorbidities** | 17.40 (11.34-26.70) | **<0.001** |
| **Other Comorbidities** | | |
| **Chronic Pulmonary Disease** | 1.26 (1.12-1.42) | **<0.001** |
| **Hypertension** | 1.25 (1.09-1.44) | **<0.01** |
| **Obesity** | 1.43 (1.26-1.62) | **<0.001** |
| **Diabetes** | 1.41 (1.26-1.58) | **<0.001** |
| **Smoking** | 1.01 (0.87-1.18) | >0.05 |
| **Surgery Level** | | |
| **Multilevel ACDF** | 1.30 (1.15-1.48) | **<0.001** |
| **Primary Surgical Indication** | | |
| **Cervical Degenerative Disorders** | 1.10 (0.78-1.56) | >0.05 |
| **Cervical OPLL** | 1.09 (0.60-1.98) | >0.05 |
| **Cervical DISH** | 3.71 (1.91-7.22) | **<0.001** |
| **Cervical Kyphosis** | 2.10 (1.11-3.97) | >0.05 |
| **Pseudoarthrosis** | 1.23 (0.86-1.76) | >0.05 |
| **Cervical Spine Fracture** | 3.15 (2.00-4.96) | **<0.001** |
| **Cervical Dislocation** | 0.72 (0.28-1.83) | >0.05 |
| **Cervical SCI** | 1.72 (0.87-3.42) | >0.05 |
| **Rheumatoid Arthritis** | 1.02 (0.79-1.31) | >0.05 |
| **Ankylosing Spondylitis** | 1.51 (0.69-3.29) | >0.05 |
| **Insurance** | | |
| **Medicaid** | 0.99 (0.80-1.23) | >0.05 |
| **Private insurance** | 0.78 (0.68-0.90) | **<0.01** |
| **Self-pay** | 1.25 (0.77-2.04) | >0.05 |
| **No charge** | 1.00 (1.00-1.00) | >0.05 |
| **Other** | 0.72 (0.56-0.93) | **<0.05** |
| **Hospital Teaching Status** | | |
| **Urban nonteaching** | 1.11 (0.78-1.60) | >0.05 |
| **Urban teaching** | 1.55 (1.11-2.16) | **<0.05** |
| **Hospital Location** | | |
| **Midwest** | 1.20 (0.93-1.55) | >0.05 |
| **South** | 1.03 (0.81-1.30) | >0.05 |
| **West** | 1.09 (0.84-1.42) | >0.05 |
| Footnote: This analysis excluded patients who had dysphagia codes documented within the first ten diagnosis fields at index admission. Model covariates are identical to Model 1.  OR: Odds Ratio, CI: Confidence Interval, ACDF: Anterior Cervical Discectomy and Fusion, OPLL: Cervical Ossification of the Posterior Longitudinal Ligament, DISH: Cervical Diffuse Idiopathic Skeletal Hyperostosis, SCI: Cervical Spinal Cord Injury  ^1^Comparisons between patients with and without dysphagia were conducted using multivariable logistic regression with p-values adjusted for multiple testing using Benjamini-Hochberg false discovery rate (FDR) method. | | |
